# Interlinking financial stability regulation and governance in German professional soccer: contribution and implications

**DOI:** 10.3389/fspor.2024.1486759

**Published:** 2024-10-24

**Authors:** Sandy Adam, Birgit Bachmaier

**Affiliations:** Department of Sport Economics and Sport Management, Leipzig University, Leipzig, Germany

**Keywords:** club licensing, financial regulation, harmonization, incentive mechanisms, independence, management and oversight, stakeholder participation, sustainability

## Abstract

**Introduction:**

This study explores the integration of financial stability regulation in professional soccer within the framework of sport governance, focusing on the German context. The research examines how financial regulations influence key governance principles such as accountability, transparency, and sustainability, while also addressing the challenges posed by the dynamic nature of professional soccer.

**Methods:**

A qualitative methodology was employed, using focus group discussions with nine experts, including representatives from soccer clubs, auditing firms and other relevant stakeholders. The discussions aimed to capture diverse perspectives on the impact of financial stability regulation on governance practices within the German soccer league and clubs.

**Results:**

The findings reveal that financial stability regulation is effective in promoting financial discipline and accountability at both league and club levels. However, the study also identifies challenges, such as the need for greater harmonization of regulatory frameworks across different levels of professional soccer and the potential benefits of implementing incentive mechanisms within the financial stability regulation to improve governance at the league and club levels.

**Discussion:**

The study underscores the importance of a multi-dimensional approach to financial stability regulation, considering political, systemic, and organizational dimensions. It highlights the potential for improving governance through the adoption of independent governance models and more practical applications of governance principles. Future research could further explore these areas, offering insights that could enhance the effectiveness of financial regulation in professional soccer and potentially other sport contexts.

## 1 Introduction

Professional sport leagues require their associated clubs to comply with various governance frameworks, with regulation being a key component. Regulation involves the establishment of rules and principles by public or private entities with the authority to influence and control the behavior of others to achieve specific objectives. This includes a system for monitoring and ensuring adherence to these rules (1, 2). In soccer, regulation covers the sporting context, including competition formats, and promotion and relegation systems. Economic regulations govern the redistribution of broadcasting rights and the sport labor market. Financial regulations have been introduced to control club spending, promote competitive balance, and ensure financial stability (3).

UEFA's Financial Fair Play, introduced in 2010 to enforce financial discipline among clubs in UEFA competitions, required clubs to avoid overdue payables and balance soccer-related revenues with expenditures. In 2022, it was replaced by the UEFA Club Licensing and Financial Sustainability Regulations, which introduced a squad cost limit, capping spending on player wages, transfers, and agent fees at 70% of club revenues by the 2025/26 season (4). Similar financial regulation exists in most European professional soccer leagues, including the German Bundesliga (3).

The German Soccer League (*DFL*) consists of the German Soccer League Association (*DFL e.V.*), which represents the 36 professional clubs in the 1st and 2nd Bundesliga, and its 100% subsidiary, the German Soccer League Corporation (*DFL GmbH*). Together, DFL e.V. and DFL GmbH are responsible for managing the sporting competition and overseeing financial stability. Clubs must comply with DFL's articles of association and league statutes, particularly the licensing regulations (*Lizenzierungsordnung*). These regulations require clubs to maintain financial discipline through security deposits, detailed financial reporting, and external audits to ensure their ability to compete in the current and following season (5).

The examination of financial crises in professional soccer has been a longstanding concern (6–11) and remains relevant today (12–15). Several studies have examined regulatory regimes aimed at preventing financial crises, such as national licensing procedures (3, 16, 17) and UEFA's Financial Fair Play (18–21). Others have explored the impact of these regimes on financial performance and competitive balance (22–24).

A research gap exists in understanding professional sport leagues’ financial stability regulation within the framework of sport governance. While such regulation provides incentives for clubs to develop governance principles that meet the set requirements, fulfilling minimum principles may not be sufficient for long-term success. Therefore, this study aims to explore how financial stability regulation impacts governance principles in professional soccer. Specifically, it addresses the following research questions:

RQ1: How can DFL's financial stability regulation be understood within the framework of sport governance?
RQ2: How does this regulation enhance governance principles at club and league levels?
RQ3: What adjustments or extensions to this regulation might be necessary to align with good governance principles?

A clear definition of key concepts, particularly financial stability and sport governance is crucial for effectively understanding and analyzing the study's inquiry. Financial stability, while central to financial regulation, lacks a universally accepted definition and is often defined more by what it seeks to prevent than by what it explicitly entails. In economics, it typically refers to the absence of negative outcomes, such as institutional collapses with significant economic and social impacts (25). In professional soccer, financial instability often manifests as operating losses, excessive debt, and insolvency, frequently driven by overinvestment in playing talent (13, 26, 27). To address these issues, this study proposes that financial stability involves prudent management to ensure current success without compromising future viability. Key indicators include profitability, liquidity, and solvency (14, 28), along with risk mitigation strategies to protect stakeholders (29) from potential supply and demand shocks such as sponsor insolvency or declining ticket sales (27).

To ensure financial stability in professional soccer, an effective governance framework must require league and club decision-makers to prioritize the best interests of their organizations and stakeholders. From this perspective, financial stability regulation intersects with three types of sport governance: political, systemic, and organizational (30). Politically, financial stability regulation helps DFL achieve broader sport-political objectives by maintaining the integrity of competitions, benefiting both soccer and society (31). Systemically, it shapes relationships between DFL and its associated clubs through mechanisms such as licensing schemes and the redistribution of media rights revenues, balancing cooperation and competition. The regulation also considers interests of other stakeholders, including fans, sponsors, media, and community organizations (32, 33) reflecting systemic governance shifts from hierarchical to network-based structures (21, 34). Organizationally, the regulation involves aspects of transparency, accountability, and ethical principles by enforcing financial reporting and auditing, preventing mismanagement, and guiding strategic financial decisions.

This study contributes to the sport economics and management literature by offering a framework that links financial stability regulation with political, systemic, and organizational sport governance. It responds to calls for exploring how regulation can influence governance principles in sport organizations (35, 36). The study underscores the evolving role of professional soccer in promoting sustainability and transparency, particularly in response to challenges like the COVID-19 pandemic. Initiatives like DFL's Taskforce Future of Professional Soccer (*Taskforce Zukunft Profifußball*) have led to the integration of sustainability criteria into licensing regulations (37, 38). As these frameworks continue to evolve, the study's findings offer important implications for enhancing governance structures within soccer and potentially other sports as well.

## 2 Conceptual framework: financial stability regulation in the context of sport governance

This study integrates Bachmaier et al.'s (17) model for evaluating financial stability regulation in soccer leagues with governance principles from academic literature and corporate and sport governance frameworks. Bachmaier et al.'s model, which includes 72 regulatory requirements across six key areas—structural basis, guarantees, documents, process, reliability and credibility, and governing bodies’ assertiveness—serves as the foundation for assessing and monitoring soccer clubs’ financial stability. Additionally, a range of good governance principles, reflected in approximately 50 sport governance frameworks, has been discussed in the academic literature by scholars such as Parent and Hoye (39).

The authors conducted a theoretical comparison between Bachmaier et al.'s financial stability criteria and governance principles from the academic literature, along with corporate frameworks like the German Corporate Governance Code (40) and sport-specific frameworks such as the Sports Governance Observer (41) and the Sports Governance Code (42). This comparison identified four key governance principles—accountability and control, social responsibility and solidarity, transparency, and governance structure—as the most relevant for establishing a robust conceptual framework guiding the empirical study. Each of these principles plays a critical role in supporting financial stability. Accountability and control are closely aligned with regulatory oversight mechanisms, ensuring that clubs are held responsible for their financial decisions and management practices. Social responsibility and solidarity focus on the need for financial practices that take stakeholders’ interests into account while promoting long-term sustainability. Transparency ensures that financial information is disclosed openly and is easily accessible, which is essential for effective monitoring and early detection of potential financial issues. Lastly, governance structure refers to the formal systems of decision-making and oversight within clubs and leagues, ensuring that financial regulations are enforced effectively and that clubs operate within clear, structured guidelines.

### 2.1 Financial stability regulation in the context of accountability and control

Accountability is a cornerstone of good governance. Grant and Keohane (43) define accountability as the right of certain actors to hold others to standards, assess compliance, and enforce sanctions if responsibilities are unmet. As a control mechanism, accountability influences organizational decision-making, ensuring that actions align with the expectations of owners, funders, and regulators (44). A key aspect of accountability is effective financial management. Morrow (33) highlights the importance of prudent resource use and fulfilling financial obligations. Financial accountability is closely linked to formal accounting standards, where transparency is demanded by owners, funders, and regulators. Organizations meet these demands through legislative, regulatory, and judicial mechanisms, including disclosure and compliance requirements (45).

Financial stability regulation intersects with financial accountability through comprehensive documentation, external audits, regulatory actions, sanctions, and independent decision-making bodies. For example, the DFL licensing process requires clubs to submit detailed financial information—such as budgets, financial statements, player contracts, and marketing contracts—and to undergo ongoing monitoring to ensure compliance with financial stability requirements. External audits enhance credibility of financial data. Additionally, clubs must prove they have no outstanding payments to staff, other clubs, or authorities, with the DFL imposing strict deadlines and rules to reinforce financial accountability. DFL uses specific criteria, such as liquidity projections, to grant club licenses and imposes penalties like point deductions, fines, and suspensions to ensure compliance. These measures encourage clubs to maintain financial discipline, protecting the league's integrity and exemplifying accountability through adherence to standards and the imposition of consequences (46).

### 2.2 Financial stability regulation in the context of social responsibility and solidarity

The concept of accountability has significantly evolved over time. Initially focused on financial responsibility to owners, funders, and regulators, it now encompasses a broader range of responsibilities. This modern perspective involves not only external oversight but also an organization's recognition of its legal obligations and “license to operate”, which considers those indirectly affected by its actions (44, 47). Sport organizations are now accountable to diverse stakeholders. Soccer clubs, for instance, must balance their economic responsibilities within their relationships with the league and other clubs. However, financial difficulties can arise from managerial errors or external factors, such as supply or demand shocks. To address these challenges, DFL has established a protection fund, supported by contributions from all licensed clubs, to assist those facing short-term financial difficulties. This mutual support system reflects a solidarity mechanism that ensures clubs’ financial stability and competitive integrity, with broader societal implications.

Soccer clubs are more than sport organizations; they are social and cultural institutions embedded in local communities (48). Consequently, clubs are increasingly expected to be accountable to the wider community. Fans are now more concerned with how clubs address issues like youth development, community engagement, and environmental sustainability. In response, DFL's Taskforce Future of Professional Soccer has prioritized integrating sustainability into its operations. This includes addressing economic, social, and ecological aspects to tackle global challenges like CO₂ neutrality and environmental protection, while also strengthening ties with fans through participatory structures and regular dialogues (37).

### 2.3 Financial stability regulation in the context of transparency

Transparency, closely tied to accountability, is a key principle of good governance. It involves making procedures and decisions clear and accessible to stakeholders. Schnackenberg and Tomlinson (49) define transparency through three dimensions: disclosure, clarity, and accuracy. Disclosure involves providing timely and open information to align organizational and stakeholder interests, clarity ensures the information is understandable, and accuracy guarantees its reliability. Rawlins (50) argues that true transparency requires organizations to actively create and disseminate knowledge, not just share information.

The growing public scrutiny of the financial dynamics of major soccer clubs—from their enormous revenues to significant debts—underscores the demand for greater transparency in the sport. Transparent financial disclosure is crucial to preserving the credibility and integrity of professional soccer. Consequently, financial stability regulation must prioritize transparency, with leagues disclosing their rules and principles to all relevant stakeholders, ensuring a clear understanding of contractual rights, financial criteria, and procedures for assessing and monitoring financial stability. Requiring clubs to publish annual financial statements on both their websites and the league's site would provide all stakeholders with current financial information, including assets, income, and cash flow. This transparency would enhance the financial stability of leagues and clubs by enabling informed stakeholder monitoring and auditing.

### 2.4 Financial stability regulation in the context of governance structure

The structured arrangement of boards and committees is crucial to governance, as it shapes the bodies responsible for safeguarding stakeholder interests, overseeing management, and making strategic decisions in sport organizations (51). Both corporate and sport governance literature have examined elements of governance structure, such as the independence of board members (51–53), CEO duality (52, 53), board size (53–55), and board diversity (55–57). For example, club financial managers and members of the league's financial control bodies must possess financial literacy and experience to ensure financial stability of clubs and leagues. Additionally, maintaining independence from league participants is essential for unbiased governance and decision-making. In this context the independence of financial control bodies is a significant aspect, as seen with the *Direction Nationale du Contrôle de Gestion (DNCG)* in French professional soccer. However, other leagues, like the DFL, face challenges due to the lack of complete independence, as board members often have ties to the leagues or clubs they regulate. Integrating these regulatory requirements with broader concepts of governance structure underscores the importance of diversity, expertise, and independence in effective governance.

By empirically examining these connections, we can better understand how financial regulation is influenced by and contributes to good governance principles in professional soccer.

## 3 Materials and methods

This study employed a qualitative design using focus group discussions to explore the interconnections between financial stability regulation and governance in German professional soccer. The goal was to gather diverse perspectives for a comprehensive understanding of the matter. A socio-constructivist approach was selected, acknowledging that knowledge is constructed through social interactions. This approach was instrumental in capturing the dynamic and contested nature of complex phenomena such as those being examined. It also encouraged reflective dialogue among participants, allowing them to engage with and challenge each other's perspectives. This uncovers deeper insights that would be difficult to obtain through individual interviews (58, 59).

### 3.1 Selection of experts and data collection

Given the specificity of the research questions, it was necessary to purposefully select experts for the study (60). As Schütz (61) defines, experts are individuals who possess complex, integrated knowledge relevant to the research problem—knowledge that is not universally accessible. In this study, the selection of experts was guided by the stakeholder approach to professional soccer. Freeman defines a stakeholder as “any group or individual who can affect or is affected by the achievement of the organization's objectives” (62: p. 46). This study aimed to identify experts from stakeholder groups whose sporting, economic, and social interests are connected to financial regulation and governance in professional soccer. Key stakeholders included members of DFL financial control bodies and representatives from Bundesliga clubs. Both groups influence and are influenced by the development and implementation of financial stability regulation. Specifically, club representatives must comply with this regulation, while DFL bodies monitor compliance. On the other hand, academics and experts, such as lawyers, contribute to the discourse but are neither directly involved in the development of the regulation nor affected by it.

The authors recruited nine experts, carefully selecting individuals well-suited to the research setting. The group consisted of one woman and eight men, reflecting the gender composition typically found in the relevant stakeholder groups, such as the club and DFL boards and committees, which are predominantly male (63). Although additional female experts were contacted during the recruitment process, they declined to participate. To maintain anonymity, participants are referred to as “P1”, “P2”, and so on. All experts had relevant qualifications and experience in finance, governance, and other pertinent areas. While officials from DFL's operational license management declined to participate, several experts had leadership experience within the DFL or the German Soccer Association (DFB), offering valuable insights. Additionally, the perspective of DFL governance was represented by current and former members of the DFL Finance Commission and the Licensing Committee, key bodies involved in DFL's financial oversight. Table 1 provides detailed background information on the participants.

**Table 1:** Background information focus group participants (*N* = 9).

Code	Focus group (FG)/Part	Gender	Previous or current job role (work experience in years)/Job position	Previous or current organization	Degree of involvement in developing or implementing financial stability regulation	Degree of influence by financial stability regulation
P1	FG1/1	Male	Finance, controlling (9)/Director	Professional soccer club	Involved	Influenced
	FG1/2					
P2	FG1/1	Male	Communication, sponsoring, and events (28)/Director	Club sponsor	Not involved	Influenced
	FG1/2					
P3	FG1/1	Male	Finance, consulting (10)/Sport business director, partner	Accounting and consulting firm	Involved	Not influenced
	FG1/2					
P4	FG1/1	Male	Research and teaching (8)/Course leader	Higher education institution	Not involved	Not influenced
	FG1/2					
P5	FG2/1	Male	Law (13)/Lawyer, partner	Law firm	Not involved	Not influenced
	FG2/2					
P6	FG2/1	Female	General management (15)/CEO	Fan organization	Not involved	Influenced
	FG2/2					
P7	FG2/1	Male	Strategy consulting in sport (2)/Director	Accounting and consulting firm	Involved	Not influenced
	FG2/2					
P8	FG2/1	Male	Commercial and financial management (7)/Board member	Professional soccer club	Involved	Influenced
	FG2/2					
P9	FG2/1	Male	Commercial rights management, business development, finance, controlling (13)/Managing director	Sport marketing company	Not involved	Influenced
	FG2/2					

In preparation for the focus group discussions, the authors consulted scientific forums, methodological literature, and three experienced researchers. This process led to the creation of a checklist and guideline, which were pretested in a research colloquium at the authors’ home university. Feedback from academics, experts, and observers, including students, was used to make minor adjustments. Before the focus group commenced, participants were informed about the study's objectives and how their data would be ethically managed. They also signed consent and data protection forms, with particular emphasis on the pseudonymization of the conversation content.

Two focus groups were conducted, each with four to five participants, over two sessions between November 16 and December 6, 2021, during the COVID-19 pandemic. A total of four online sessions, averaging 96 min each, were moderated by the authors. Participants were instructed to adopt an objective “bird's-eye view” perspective. Following a thematic introduction on financial stability and sport governance, a hypothesis from Dietl and Franck was presented: “The (partly hidden) financial crisis in German [soccer] is caused by substantial governance failures” (7: p. 668). Although somewhat dated, this quote remained relevant during COVID-19, effectively stimulating discussion and serving as a foundation for progressing through four thematic areas. These themes were based on the conceptual framework and guided by prompts, main questions, and supplementary questions.

After each session, the authors generated postscripts to capture initial impressions. Sessions were recorded and transcribed verbatim with pseudonymization (64). Both authors transcribed and reviewed the material using MAXQDA Analytics Pro 2022 software, resulting in 89 pages of text and 383 minutes of conversation. Transcripts and quotes were sent to participants for review and approval (member checking). While all transcripts were analyzed in their original German, quotes were translated into English.

### 3.2 Data analysis

A qualitative content-structuring analysis of the focus group transcripts was applied, following the method outlined by Kuckartz and Rädiker (64) and utilizing MAXQDA Analytics Pro 2022 software. Initially, both authors independently familiarized themselves with the dataset, which consisted of four transcripts. This process involved reading the transcripts, marking important passages, and writing memos to capture initial analytical ideas. A total of 526 memos were created, forming the basis for developing preliminary categories.

Subsequently, the authors conducted a joint analysis, reviewing the transcripts passage by passage while incorporating the earlier memos. This collaborative review resulted in a high degree of agreement on the memos and preliminary coding ideas. Initial categories were developed deductively, based on the conceptual framework and focus group guidelines, with text segments systematically assigned to these categories. During the coding process, additional inductive categories and subcategories emerged. To ensure rigor, the authors referenced Thompson et al.'s (65) systematic review on good governance principles, which was published while the data were being analyzed. They applied a “theoretical labeling” process, which involved retrospectively amending initial inductive categories based on new theoretical insights. While the term “theoretical labeling” is not widely standardized, the concept aligns with grounded theory and the practice of integrating emerging theory and literature into the coding process after initial data-driven coding (66, 67).

Given the complexity of the topics discussed and the extensive cross-referencing between transcripts, the authors intentionally employed a circular consensual coding approach to ensure a comprehensive and nuanced analysis. This method diverges from the more commonly used approach outlined by Kuckartz and Rädiker (64), where coding is first conducted independently by multiple researchers, followed by a comparison and consensual discussion of the resulting categories. Instead, the authors opted for a joint analysis and discussion of each text segment throughout the entire coding process.

This method aligns with the socio-constructivist approach of the study, which emphasizes the co-construction of knowledge through interaction and discussion. By conducting coding collaboratively, the authors ensured that the process was not merely about achieving consistency in coding but about fully exploring the depth and richness of the data in a dynamic, collaborative manner. The continuous reflective dialogue between the coders allowed for real-time cross-verification of insights, resolving ambiguities immediately and reducing potential bias or misinterpretation of the data. This approach enhances credibility and dependability by promoting rigorous validation of the findings through peer interaction, ensuring that emerging themes are well-grounded in participant perspectives and reducing the risk of individual researcher bias (64, 68). This method aligns with established qualitative practices, such as those outlined by Patton (60) and Fereday et al. (66), who advocate for flexible and iterative approaches when coding complex data.

Throughout this process, starting with deductive coding and evolving through the development of inductive and theoretically labeled categories, the authors maintained rigorous documentation in the form of theoretical memos. These memos detailed the characteristics of each category, including definitions, anchor examples, and coding rules, ensuring that the evolving category system was both methodologically sound and theoretically informed. This transparent documentation process not only contributes to the confirmability of the study but also provides a clear audit trail, allowing for external verification and reducing subjectivity in the analysis. The final category system, described in the subsequent section, consists of four main categories and 110 subcategories, divided into 45 deductive, 51 inductive, and 18 theoretically labeled categories.

## 4 Results

This section presents the main categories, categories and subcategories that emerged from the data analysis, accompanied by quotations, detailed explanations, and contextualization. In addition to addressing RQ1, this section provides insights into how DFL's financial stability regulation enhances governance principles at the club and league levels (RQ2) and explores necessary adjustments and extensions to align the regulation with good governance principles (RQ3).

### 4.1 Professional soccer governance system

The experts discussed various issues specific to the governance of professional soccer in Germany. These aspects are crucial as they directly impact DFL's financial stability regime, with the most significant elements summarized in Table 2.

**Table 2:** Professional soccer governance system.

Category	Subcategory	Quote
League governance structures and processes		“How do we determine these minimum requirements? It works like this: 36 clubs cast their votes on which regulations they want to adopt and which ones they do not…” (FG1/2, P1).
50 + 1 rule		“The 50 + 1 rule indeed allows for some flexibility when it comes to financing options…” (FG1/1, P4).
Conflicting goals		“…the interests are sometimes so divergent and distant from each other…” (FG2/1, P6).
Sporting and economic performance	European model of sport	“…then this overspending, it's all about just getting by somehow, no matter what happens…” (FG2/1, P6) (“Overinvestments”).
	Revenues	
	Media revenue redistribution	
	Overinvestments	
	Planning uncertainty	
Cultural asset		“…‘too popular to fail’. It's too much a cultural artifact, too much reputation, too strong a brand, too many fans, and immense importance behind it” (FG2/2, P7).

League governance structures and processes were a primary focus, particularly the decision-making procedures surrounding modifications to DFL's financial stability regulation. While this governance system offers a democratic approach, it also reveals conflicting goals during negotiation processes, especially between individual clubs’ interests and the collective interests of the league. As one participant emphasized:

“When it comes to defining what [solidarity] should encompass and determining how much clubs are willing to compromise, give up certain things for the greater good, or redistribute money, that's when the doors close again” (FG2/1, P6).

The 50 + 1 rule is a structural peculiarity in German professional soccer. This rule ensures that clubs retain majority control over their professional teams by requiring the member's association (*eingetragener Verein, e.V.*) to hold at least 50% plus one share of voting rights. Participants debated the rule's advantages and disadvantages, which are well documented in academic literature (69, 70). Some experts noted its impact on club governance structures and processes, particularly in the context of legal form choices, such as member's associations vs. corporations, which directly affect financial decisions. For instance, member's associations often struggle with building financial reserves due to restrictions imposed by German tax law on retaining financial surpluses—a restriction that does not apply to corporations. However, the 50 + 1 rule was also viewed positively as a mechanism that promotes stakeholder participation, particularly from club members and fans.

The relationship between sporting and economic success emerged as another critical aspect. Experts noted that the pyramid structure and intensity of competition are key elements of the European Model of Sport (71). The discussion on media revenue redistribution highlighted how rank-dependent revenue disparities negatively affect solidarity, driving escalating investments in player talent to achieve competitive success, which can jeopardize clubs’ financial stability. This issue also ties into the unpredictability of sporting and economic success, leading to significant planning challenges in the short, medium, and long term. Overall, the experts emphasized that further development of DFL's financial stability regulation must account for the international context, particularly as some clubs participate in European competitions like the UEFA Champions League, while others compete solely in national leagues, impacting their overall financial situation.

Finally, the experts described German professional soccer as a cultural asset. While this status fosters strong identification between fans, clubs, and communities, it may also incentivize irresponsible financial behavior. This phenomenon, known as the “soft budget constraint” (72) in sport economics, leads clubs to overspend, relying on external sources to cover deficits (73). These cultural dynamics add another layer of complexity to the governance challenges, suggesting that regulatory measures must balance the preservation of soccer's cultural significance with the enforcement of financial discipline.

### 4.2 Financial stability regulation

The participants understood financial stability regulation as a complex phenomenon, detailed in Table 3.

**Table 3:** Financial stability regulation.

Category	Subcategory	Quote
Justification		“It's a self-regulated regime. The clubs have devised it on their own to safeguard themselves from members who might violate the rules…” (FG1/2, P4).
Purpose	Operational continuity	“I also think it's important to strengthen the focus on sustainability even further in that context” (FG1/2, P2) (“Sustainability”).
	Integrity of the sporting competition	
	Image and marketability of the league	
	Sustainability	
Independence of legal form		“…this requirement to disclose information. I know of an association where they found it quite frustrating because they don't like discussing the organization's economic success as a registered entity…” (FG1/2, P4).
Regulatory strategy		“If you do something extra…then you should think about a bonus system.” (FG1/2, P1).
Enforceability		“…then you would need to be consistent in making sure that clubs are also removed…” (FG2/2, P9).
Continuous improvement	Involved stakeholders	“…I believe it's crucial to either continue the task force or bring in external experts who offer a different perspective or contribute additional expertise” (FG2/2, P6) (“Involved stakeholders”).
	Incentive problems	
	Harmonization	
	Credibility	
	Current trends	
	Independent decision on eligibility to participate in the sporting competition	

First, they highlighted that DFL's financial stability regulation is justified as part of a self-regulatory regime developed and monitored by the 36 professional soccer clubs, which are the sole members of DFL e.V. Their legitimate interests are reflected in the regulation's purpose, including preventing insolvencies to ensure operational continuity, maintaining the integrity of the competition, and enhancing the Bundesliga's image to boost marketability, both nationally and internationally. Experts also advocated for expanding the regulation's purpose by encouraging clubs to guide their business conduct based on sustainability principles.

DFL's financial stability regulation applies uniformly to all clubs, regardless of their legal structure. While this promotes consistency throughout the league, it also presents challenges. Member's associations, which emphasize democratic processes, may struggle to comply with these regulations due to the need for broad member approval before securing external funding or making swift financial decisions. In contrast, corporations, with more efficient decision-making and better access to capital, can more easily navigate the regulation. This disparity highlights the need for a nuanced approach that considers the varying capacities of clubs while ensuring financial stability across the league. DFL employs different regulatory approaches to influence club behavior. Mandatory provisions, such as maintaining positive equity, come with sanctions if not complied with, ensuring financial accountability. Additionally, there are provisions, such as sustainability criteria, that are not sanctioned. The experts suggested that DFL's regulatory strategy could benefit from incorporating broader incentive mechanisms (see Section 4.4 for more details).

Enforceability was discussed as a contested space between consistently imposing rules and allowing situational flexibility, particularly during league-wide crises. This tension involves balancing good governance principles like credibility and integrity with commercial considerations, including the league's marketability. Some experts argued that, for opportunistic reasons, a league might retain financially struggling clubs with strong brand value or fan bases despite regulatory shortcomings to protect growth opportunities. Others pointed to external shocks that may necessitate flexibility. One participant heavily criticized DFL's flexible enforcement during the COVID-19 pandemic, stating:

“…we’ve been saying that everything is improving and becoming stricter yet at the same time, we’re halting the licensing process. That doesn’t seem credible” (FG1/2, P1).

In further developing the regulation, experts recommended involving various stakeholders, including club officials, league bodies, fans, and independent third parties, to ensure good governance. It is crucial to consider current trends and developments, such as sustainability and innovation, to keep the regulation relevant and effective. Experts identified several challenges to implementing these enhancements, including the league's governance structure and processes, inherent planning uncertainty, and concerns about the credibility of such interventions. They also emphasized the importance—and difficulty—of harmonizing financial stability regulations with UEFA's Club Licensing and Financial Sustainability Regulations and DFB's regulatory framework for the 3rd league.

Finally, experts debated whether independent decisions should be made in assessing and monitoring financial stability or if independent actors should be involved. Some advocated for this approach, emphasizing objectivity and independence:

“Perhaps a model like that in France is conceivable. There is an external licensing body; this approach automatically increases credibility by ensuring that decisions are based on objective criteria” (FG2/2, P7).

However, others argued that the league and its teams possess the necessary expertise:

“Just as I and many others see the league as a collective team product, I believe it should be up to the teams themselves to assess the economic stability and financial risks that individual clubs face. No one understands the inherent difficulties within the financial system of a German league better than the actors in the league themselves” (FG1/2, P4).

### 4.3 League

The following subsections discuss the findings on league governance structures and processes. To provide context, it is essential to briefly outline the structure and procedures of DFL's financial control bodies. According to Annex X of DFL's licensing regulations, clubs must submit documents to DFL GmbH by specific deadlines. License managers review these submissions and recommend decisions based on financial stability. A management committee of three to five members evaluates these recommendations and may impose conditions. If conditions are imposed or a club appeals, the licensing committee, consisting of six members elected by DFL e.V.'s general assembly, reviews the case (5).

#### 4.3.1 Governance structures

Governance structures encompass all bodies responsible for financial control and include elected governance bodies like the licensing committee, as well as DFL's operational management, particularly the licensing directorate and its managers (see Table 4).

**Table 4:** League governance structures.

Category	Quote
Responsibilities	“License manager 1, who is responsible for operations, so to speak, organizes the licensing process, oversees its execution, and then provides recommendations…” (FG1/2, P3).
Caliber	“…ultimately, you can consider appointing neutral people who are especially suitable for this role due to their expertise” (FG2/2, P6).
Independence	“…you might now question, from a good governance perspective, whether it's truly appropriate to have club representatives on the licensing committee” (FG1/2, P1).
Tenure	“…I would say that license manager 1, who has been in charge of this for over 25 years, approaching 30 years, holds a position that, in a way, makes him somewhat immune to criticism…” (FG1/2, P4).
Size	“I have great respect for license manager 1, but none of us are immune to health issues that could prevent us from working every day, and the risk is simply too high” (FG1/2, P1).

Focus group discussions revealed that license managers contribute positively to the league's governance through their expertise, extensive experience, and the efficiency afforded by a small team. However, some participants raised concerns about the centralization of license management among a limited number of individuals. This concentration of power could compromise independence and increase the risk of inaccuracies that may jeopardize the process. Consequently, experts suggested incorporating external control bodies to enhance the system's integrity:

“I also see independence there. From examples, I understand the significance of establishing an external body” (FG1/2, P1).

The lack of independence and insufficient financial expertise within the licensing committee, consisting of elected club representatives who are not required to have financial backgrounds, raised notable concerns among experts. As a result, they advocated for the inclusion of independent members from licensed professions, such as chartered accountants:

“In the interest of good governance, I would recommend establishing a committee alongside DFL that consists of non-club representatives. The primary selection criterion should be financial training and experience” (FG2/2, P3).

#### 4.3.2 Governance processes

Governance processes are closely intertwined with governance structures, with each exerting influence over the other. Table 5 provides a summary of the specific subcategories discussed in this subsection.

**Table 5:** League governance processes.

Category	Subcategory	Quote
Oversight		“Until the final decision is made, license manager 1 has full autonomy, however, when the decision progresses to the second instance the licensing committee steps in. The DFL supervisory board is not involved in this process” (FG1/2, P4).
Financial control body processes	Decision-making processes	“…I prefer not to be in a situation where I have to decide about another club while serving as a club representative on the licensing committee” (FG1/2, P3) (“Dual mandates”).
	Conflicts of interest: dual mandates, exclusion from voting rights	
	Succession planning	
Stakeholder participation	Forms of participation	“…how could one possibly achieve true solidarity, which would essentially require a complete overhaul of the European Model [of Sport], a model that was originally founded on competition” (FG2/1, P5) (“Solidarity”).
	Principles of participation: inclusiveness, solidarity	
Transparency	Transparency in the process of assessing and monitoring financial stability for competition eligibility: medium, concerned parties, decision	“You can clearly see the requirements that clubs must meet, the documents they need to submit, and the formats they must use to present their information” (FG1/2, P3) (“Decision”).
	Create and increase transparency	

With regard to the oversight mechanisms for DFL's financial control bodies, experts criticized the lack of a specialized body overseeing DFL's license management, which could lead to unchecked authority and potential conflicts of interest when assessing the financial stability of clubs. This issue is closely linked to the previously identified concentration of power within the league's governance structures.

Conflicts of interest emerged as a critical concern, particularly those stemming from the dual roles of club representatives serving on the licensing committee. Conflicts arise when committee members are responsible for reviewing the financial stability of clubs. While the rule in Section 18(4) of DFL e.V.'s statutes, which prohibits committee members from voting when their own club is involved (5), is seen as a positive governance measure, experts emphasized the lack of broader rules addressing conflicts from dual mandates. Notably, such conflicts can create moral dilemmas for licensing committee members. Consequently, experts urged adjustments, such as involving independent bodies in decisions regarding club eligibility for DFL's competitions based on financial considerations.

The extent to which stakeholders beyond the clubs and the league should participate in decision-making processes concerning financial stability was another area of debate. Stakeholders considered included players, fans, club members, soccer associations, media, sponsors, and public authorities. One expert, representing a fan organization, advocated for the inclusion of fan representatives in consultations:

“This isn’t about having a veto right at all but at the very least, there should be consultation, as there are numerous other options available…” (FG2/2, P6).

This call for consultation is consistent with moderate forms of participation, as outlined in Arnstein's (74) typology of citizen participation, which suggests that consultation, while not granting decision-making power, allows for valuable stakeholder input.

Solidarity among clubs was recognized as a crucial principle of participation, whether voluntary or regulated through mechanisms like default insurance or a protection fund, as outlined in Sections 5(9) and 8(8b) and Annex VIII of DFL's licensing regulations (5). However, opinions on this issue were divided. According to experts, some clubs were reluctant to support solidarity measures that could cushion competitors during crises, fearing these measures might encourage risky overinvestments. As one participant noted:

“It doesn’t seem right that we should receive less TV revenue just to keep an overconfident competitor afloat…” (FG1/1, P4).

Conversely, there was strong support for league-wide solidarity regulations aimed at mitigating external shocks:

“It shouldn’t be the responsibility of an individual club to protect itself against extreme crises. Instead, other institutions, like a league organization, should take appropriate measures” (FG2/1, P8).

This support is particularly relevant given the financial challenges some clubs face due to their member association structure and the 50 + 1 rule central to German professional soccer.

Experts generally rated the transparency of DFL's processes for assessing and monitoring financial stability for competition eligibility as high. This assessment was reflected in the media used, the communication with concerned parties, and the clarity of decision-making.

### 4.4 Clubs

This section outlines the findings on the interconnections between financial stability regulation and governance at club level.

#### 4.4.1 Governance structures

In the context of governance structures, summarized in Table 6, experts discussed the roles of management and oversight bodies in maintaining clubs’ financial stability.

**Table 6:** Club governance structures.

Category	Subcategory	Quote
Management and oversight structures	Oversight: internal, external	“You need to address the issue of club supervisory boards first; everything else is merely a consequence of that” (FG1/1, P1) (“Internal oversight”).
	Composition: responsibilities, caliber, independence, diversity, tenure, size	“For instance, one could require that [supervisory board] members are able to analyze financial metrics and have a background representing the members’ interests…” (FG2/2, P8) (“Responsibilities”, “Caliber”, “Diversity”).
Nomination committee		“The leverage is that you have a nomination committee responsible for finding qualified candidates…” (FG1/2, P1).
Annual general meeting		“Given the democratic framework of German soccer, we believe that at the very least, the general meeting provides fans with a platform to voice their opinions” (FG2/2, P6).
Advisory board		“There are also very productive discussions taking place, for example, through committees outside of the main structure, such as an economic advisory board, which is crucial for bringing in specialized knowledge” (FG2/2, P9).

Management structures, which include elected bodies such as the management board and relevant departments like finance and controlling, are essential for club governance. Oversight structures, including the supervisory board, league-level financial control bodies, and third-party auditors, play a crucial role in aligning management's decisions with club goals and league expectations. Experts emphasized that oversight structures are vital for preventing power concentration and demanded the establishment of a mandatory supervisory board to enhance club governance. They also highlighted the importance of external auditors, noting that existing rules including specific plausibility checks, particularly those related to transfers, are critical for financial stability.

Experts stressed the need for clearly defined domain responsibilities and caliber for the composition of management structures. One expert suggested:

“Binding guidelines for the management board could be established, specifying that at least two or three people should be in leadership roles. This would ensure that one person focuses on match operations, while another manages finances, thereby enhancing the overall effectiveness with input from various experts” (FG2/2, P5).

Similarly, the supervisory board should have well-defined responsibilities, particularly in monitoring the management board's activities. To fulfill these roles effectively, experts emphasized the importance of having board members with appropriate qualifications, experience, and diverse backgrounds. Integrity was also identified as a crucial governance principle, with experts agreeing that board integrity is enhanced when members possess the necessary qualifications, adhere to high standards of professionalism, and are motivated to fulfill their roles.

The general meeting was highlighted as a democratic structure allowing club members to participate in decision-making, particularly during elections. However, some experts criticized this mechanism, noting that elections should not be influenced by power or “political” affiliations—an issue commonly observed in professional soccer organizations. Instead, the process should be guided by clear requirements for board positions. As one expert noted:

“What are the minimum qualifications required for these roles? It's important to seek out the right candidates, rather than choosing individuals who can quickly persuade 800 people with their personality in a brief presentation, as it's impossible to form a meaningful assessment of someone in just 3 minutes” (FG1/2, P1).

To address this concern, experts underscored the regulatory need for a nomination committee to ensure that candidates for management and oversight structures meet minimum requirements. This would help prevent conflicts of interest, ensure the caliber of individuals serving on governance structures, and support prudent financial management.

#### 4.4.2 Governance processes

Experts discussed internal and external accountability as the primary procedural link between financial stability regulation and club governance. Accountability, which encompasses financial management, reporting, and responsibility, is central to this connection (see Table 7 further below for a detailed overview).

**Table 7:** Club governance processes.

Category	Subcategory	Quote
Accountability	Financial management (Detailed illustration in Table 8)	“That's why it's even more crucial to carefully prepare these business decisions and present them transparently ensuring that I have robust financial reporting within the organization” (FG1/1, P3) (“Reporting”).
	Reporting	
	Responsibility	
Oversight	Club	“…a club once again risks everything on a single strategy despite facing relegation. Then, when they fail to remain in the league. It leads to insolvency. The real question is: what role did the other committees play in this? The supervisory board and similar bodies also agreed to the management's decisions for years” (FG2/1, P9) (“Club”).
	League	
	Third-party	
Board and committee processes	Decision-making processes	“…there's a conflict of interest because you’re serving a supervisory board while also handling tasks for a management board, raising the question of how objectively you can then conduct audits on behalf of the supervisory board” (FG2/2, P5) (“Dual mandates”).
	Conflicts of interest: code of conduct/Code of ethics, dual mandates, term limits, veto rights, pool of auditors	
Stakeholder participation	Forms of participation: consultation, co-determination, decision-making power	“…I would argue that formal participation of organized fan groups in the club should be part of the licensing process, it's not there yet…from our perspective, it's crucial for preserving a certain type of soccer…” (FG2/2 P6) (“Co-determination”).
	Principles of participation: Inclusiveness, Stakeholder-orientated communication	
Transparency	Transparency of financial information: medium, concerned parties, disclosed contents, controversy	“…the DFL has been publishing key figures for the past 3 years. I believe these figures are highly informative and offer a great deal of transparency. They are accessible online. In my opinion, everything is available, including equity ratios, revenue, profit, and loss details” (FG2/2 P8) (“Transparency of financial information”).
	Create and increase transparency: transparency through processes, Transparency through disclosed contents	

Financial management involves the planning, management, and control of a club's financial stability and is reflected in core documents and processes based on financial data and projections. This topic was thoroughly discussed during the focus group sessions and is summarized in Table 8.

**Table 8:** Accountability: financial management.

Subcategory	Quote
Multi-period view	“…incorporating more long-term planning into the licensing process would encourage clubs to operate more sustainably…” (FG2/2, P8).
Intra-year view	“…it's precisely in February, March, and April, when liquidity becomes especially tight, and every controller must inform their committees monthly – bringing us to the issue of governance – exactly how much financial leeway remains” (FG1/1, P3).
Key figures	“What is the argument against requiring equity ratios higher than zero – say, closer to 25% or 30% – especially since this crisis has demonstrated the importance of a solid equity base? Clubs with adequate equity during this period were able to absorb losses without issue; others found themselves in a much more precarious position” (FG1/1, P3).
Risk management	“…prudent risk management, which can be made a mandatory requirement” (FG2/2, P9).
Scenario planning	“…of course, promotion and relegation is an issue. However, I can still factor that into my planning, allowing me to anticipate the potential shortfall next year if relegation occurs, giving me a tool for medium-term management” (FG2/1, P8).
Accrual accounting	“…I have an invoice due by June 30. If I simply extend the due date beyond June 30, based on past experiences, and DFL has accepted that the payment still needs to be properly accounted for and settled…such tactics are not beneficial” (FG2/1, P9).
Budget revisions	“…many controllers are almost in despair when the management board and supervisory board approve yet another supplementary budget. It renders all their careful cash flow planning useless, as they must start recalculating everything from scratch” (FG1/1, P1).

Experts expressed concern that DFL's focus on financial stability for the current and upcoming season might lead clubs to prioritize short-term liquidity over sustainable financial management. In this context, they warned that clubs could exploit stakeholders as financial remedies. A managing director of a sport marketing firm illustrated this issue:

“When it comes to advance payments, which ultimately only boost liquidity they tend to be spent increasingly and then merely remain as balance sheet items. Sustainable management would involve handling this liquidity responsibly potentially over a period of 2–3 years because a marketer should not be seen as an ‘Ersatzbank’ that covers any short-term liquidity gaps” (FG1/1, P9).

The term *Ersatzbank* is a clever wordplay in German with dual meanings. Literally, it refers to the substitute bench in soccer, where players wait to replace those on the field. Metaphorically, *Ersatzbank* suggests a substitute financial institution. The humor lies in this double entendre, as it likens sport marketers or other stakeholders to a substitute bench that clubs might rely on in financial emergencies.

Experts recommended incorporating multiple time periods into financial planning to better assess and ensure clubs’ financial stability and emphasized correctly allocating income and expenses. They acknowledged the challenges of long-term planning due to the uncertainties inherent to professional soccer and highlighted scenario planning as a key management tool. This involves preparing for outcomes like promotion, relegation, or qualification for other competitions. Additionally, they stressed the importance of risk management—identifying, assessing, avoiding, and mitigating risks—as crucial to financial stability. Experts also noted that liquidity bottlenecks often occur in the season's second half and recommended mid-year analyses to ensure financial stability. These analyses would enable short-term target/actual comparisons and timely corrective actions. Focus group sessions therefore discussed financial stability indicators—covering future, present, and past metrics—including liquidity and equity measures used in DFL's licensing process, as well as profitability and insolvency indicators like the *Z* score (28) and sustainability metrics.

According to Annex IX of DFL's licensing regulations, clubs must maintain positive equity, with improvement mandates and sanctions imposed for non-compliance (5). The proposal to implement a regulated equity ratio for all clubs, including transitional periods for newly promoted teams, sparked debate among experts. Supporters argued that it could help mitigate losses, while opponents cautioned that budget adjustments through supplementary budgets could quickly erode equity, calling for stricter regulation, questioning:

“…‘balanced budget: yes/no’ or ‘supplementary budgets: yes/no’. ‘Are any losses acceptable at all?’ ‘And if losses do occur, the necessary financing must be secured’” (FG1/1, P1).

Participants supported incentivizing compliance with target figures as an extension of DFL's financial stability regulation. One participant suggested:

“Compliance with plan quality should definitely be introduced as a new measure in the licensing procedure and particularly rewarded via TV money” (FG1/1, P1).

These approaches would enhance the regulations’ stronger emphasis on sustainability and influence the mandates of auditors, as well as the roles of management and oversight structures in ensuring financial stability and good governance.

In addition to financial management, this subsection will address reporting and responsibility, as relevant aspects of governance processes (see Table 7).

Experts emphasized the importance of both external and internal reporting for planning, managing, and monitoring financial stability within clubs. External reporting involves sharing financial information, such as annual statements, with stakeholders like tax authorities and club members, while internal reporting includes management reviews and financial planning. To ensure good governance, a three-step reporting process is recommended: (1) from the finance department to the management board, (2) from management to internal oversight bodies, and (3) from these bodies to the DFL's financial control bodies. Monthly reports from operational levels to oversight bodies are also crucial, as one participant noted:

“…for the committees, for governance, I believe it would be beneficial to establish regular reporting and a more tightly integrated reporting system” (FG1/1, P3).

Experts emphasized the importance of clubs taking economic, social, and environmental responsibility toward stakeholders as a key governance principle. They warned against opportunistic management driven by the “soft budget constraint”, which could be curbed through stronger regulation. As one expert noted:

“It's always a matter of whether it's appropriate behavior to take a risk, knowing that another stakeholder might have to deal with the consequences. Maybe it's also part of corporate governance to avoid such situations if you don’t want this to happen” (FG2/2, P7).

To enhance clubs’ responsibilities, experts discussed three avenues aligned with improving DFL's financial stability regulation: strict regulation, incentivization with direct rewards, and incentivization without direct rewards.

For the strict regulation approach, experts proposed introducing a mandatory sustainability quota and long-term financial management and reporting practices. One participant noted:

“I suggest that a certain percentage – whether it's one, five, or another amount – of revenues should be allocated to social, charitable, or sustainable projects. However, if these matters aren’t centrally regulated, the tendency will naturally be to prioritize having more funds available for soccer” (FG2/2, P8).

For incentivization with rewards, experts suggested linking sustainable investments to the redistribution of media revenues. As one participant affirmed:

“Clubs need to be willing to allocate a portion of their TV revenue to invest in these sustainable projects in a clear and organized manner over the years” (FG1/1, P1).

For incentivization without direct rewards, experts recommended guidelines for sustainable projects and investments to motivate clubs to explore alternative business models, like youth development and strategic partnerships. This approach would help clubs diversify risks and build a sustainable financial foundation.

Experts emphasized the crucial role of oversight bodies in ensuring financial stability through responsible monitoring, utilizing checks and balances in line with good governance principles. Addressing and managing conflicts of interest was a key governance issue related to board and committee processes. Experts highlighted potential conflicts from the personal, sporting, and economic interests of management and supervisory board members. One participant noted:

“There's only one rule: the president and supervisory board must be re-elected. And when things are going poorly in sport, long-term planning can quickly be abandoned” (FG1/1, P1).

This quote presents an agency problem, reflecting earlier concerns about the political dynamics within clubs, where incumbents prioritize short-term success to improve re-election chances, often at the expense of long-term economic prospects. As a remedy, experts recommended introducing a mandatory code of conduct or ethics within DFL's financial stability regulation. This would help establish club-specific guidelines on ethical principles and promote responsible behavior among those managing their club's economic and financial health.

Dual mandates emerged as a significant conflict of interest during focus group sessions. Section 4(4) of DFL's licensing regulations prohibits individuals from serving on boards of multiple clubs (5) to prevent conflicts of interest. However, auditors often face dual mandates. On one hand, they are responsible for auditing financial statements, a task that involves overseeing the management board's work on behalf of the supervisory board. On the other hand, they also advise club management during the preparation of licensing documents to be submitted to DFL. This dual role creates a conflict, as auditors essentially oversee their own activities. Experts suggested critically assessing whether these roles should be separated between independent auditors to enhance governance. However, this separation should only be pursued if the benefits of increased oversight outweigh the associated costs for clubs and the league. One participant noted:

“This separation can certainly be implemented and may be beneficial from a compliance perspective. On the other hand, the synergy effect is the clear advantage of having it done from a single entity” (FG2/2, P5).

As a safeguard against competing interests and operational blindness, experts recommended that the league consider limiting or rotating the terms of management and supervisory board members. This approach could also apply to external auditors, enhancing board efficiency and preventing biased audit opinions. However, experts acknowledged that rotating auditors might be challenging due to the limited number of auditors available in professional soccer and the potentially high costs involved. They believe that DFL's financial control bodies’ right to veto the appointment of auditors, as provided in Section 8(1.1) of DFL's licensing regulations (5), is a key governance tool, allowing the league to intervene if competing interests arise. To further mitigate conflicts, they suggested creating a pool of DFL-approved auditors from which clubs must choose. One expert noted:

“There are a set number of certified auditors, and clubs are required to choose one of them for the licensing process. This approach helps prevent clubs from relying on a local business partner who might produce a favorable audit out of courtesy” (FG2/2, P8).

Focus group discussions explored the involvement of stakeholders in club decision-making on financial stability. Consultation allows stakeholders to provide input without the assurance of implementation, while co-determination involves stakeholder voting in financial decisions. In the highest form, decision-making power, stakeholders hold exclusive authority (74). According to Section 5(11) and Annex III of DFL's licensing regulations, clubs are required to organize fan dialogues (5). However, experts advocated for deeper fan involvement beyond consultation, suggesting that fans should have more responsibility in decision-making. Building on this, the experts emphasized the importance of a diverse supervisory board and called for the mandatory inclusion of a fan representative. They argued that this would contribute to a more effective board, especially when financial decisions are at stake.

Transparency is closely tied to stakeholder participation and accountability, with both internal and external aspects. Internally, it involves making key documents and processes accessible for overseeing financial stability. This has already been discussed in relation to club and league governance structures, as well as financial management and reporting. Therefore, the focus now shifts to the external aspect of transparency.

The disclosure of club-related financial information is crucial for stakeholders to evaluate clubs’ financial position. While the German Federal Gazette (*Bundesanzeiger*) is the legally mandated platform for publishing annual financial statements, its user-friendliness and accessibility were questioned. A fan organization representative noted:

“You can communicate with people much more directly, and most are now accustomed to receiving information quickly or very directly. Having to log into the Bundesanzeiger might be more of a hurdle” (FG2/2, P6).

Experts also discussed the requirement for clubs to disclose key financial figures in DFL's economic report, which is published annually on DFL's website, as outlined in Section 8(8k) Annex VIIb of DFL's licensing regulations (5). Recognizing that this information is accessible to all interested stakeholders, experts viewed it as a contribution to good governance. However, they stressed that the publication should be limited to past financial information, as releasing budget figures might compromise the clubs’ competitive position in the league. One participant remarked:

“Of course, transparency can only be retrospective, which makes sense. When it comes to budget figures for the upcoming season, I must admit I would have reservations and would be hesitant to make that public” (FG2/2, P8).

Experts held differing views on how financial data should be tailored to specific target groups. Some believed that the economic report with its key figures is sufficient for the public. As one expert commented:

“I think many people are interested, and I also think it's presented in a reasonable way, allowing anyone who wants to delve deeper to do so” (FG1/2, P2).

However, others viewed the financial data as more relevant to a specialized audience, with one expert noting:

“There are far too few people who know how to interpret such information. So, what's the point of transparency if hardly anyone can draw conclusions from it” (FG1/2, P4)?

While the general meeting was seen as a key platform for sharing financial information with members, the necessity of providing more detailed information was questioned. One expert observed:

“When it comes to transparency for fans and members I think of the general meeting. I’ve noticed there's little demand for more in-depth information, so there doesn’t seem to be a strong interest in exploring it further” (FG2/2, P8).

## 5 Discussion and conclusions

In the discussion that follows, it is explored how DFL's financial stability regulation fits within sport governance, addressing its impact on accountability, transparency, and sustainability. The regulation interacts with various stakeholders and enhances governance at both club and league levels through strict accountability and long-term financial planning. Furthermore, necessary adjustments to align the regulation with good governance principles are discussed, ensuring both immediate and long-term financial stability in German professional soccer.

### 5.1 RQ1: how can DFL's financial stability regulation be understood within the framework of sport governance?

**Figure 1:**
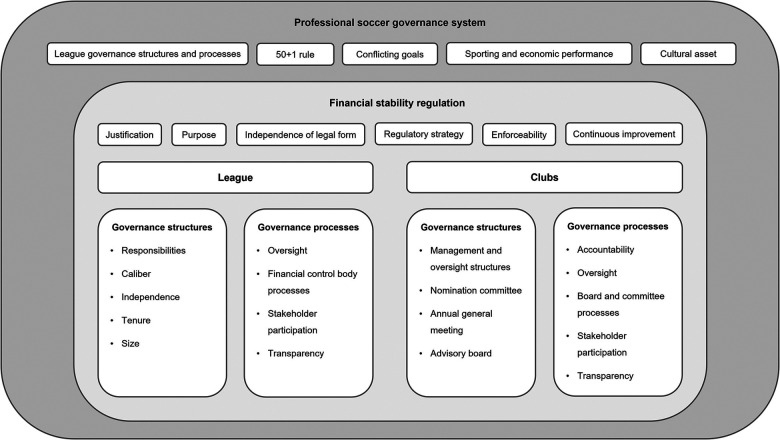
DFL’s financial stability regulation within the framework of sport governance.

DFL's financial stability regulation is part of a complex, multi-dimensional sport governance framework, which consists of three key layers shaped by the political, systemic, and organizational dimensions of sport governance (30). These layers are illustrated in Figure 1.

The regulation is embedded within the professional soccer governance system as the first layer, which involves distinct structures, processes, and a variety of actors, primarily DFL and Bundesliga clubs. Other key stakeholders include soccer governing bodies (DFB, UEFA, FIFA), media networks, fan communities, and public institutions. A significant element of this governance system is the 50 + 1 rule, which creates a unique dynamic for DFL's regulatory regime. The close relationship between sporting and economic performance further complicates this dynamic. While soccer's role as a cultural asset brings many benefits, it also intensifies these challenges by encouraging a “too-popular-to-fail” mindset. This can lead clubs to overspend with the tacit assumption that external stakeholders will step in as a financial safety net, or *Ersatzbank*, during times of crisis.

Political and systemic governance are evident in the cooperative structures between DFL and Bundesliga clubs, which voluntarily adopt regulation to ensure financial stability, reflecting the second layer. This reflects a commitment to governance that serves the DFL's legitimate interests. The regulatory strategy includes sanctions for violations but must balance strict enforcement with flexibility, especially during crises. Research suggests that elite clubs often exert considerable influence in regulatory processes, potentially leading to rules that disproportionately impact smaller clubs (15, 20). The challenge, therefore, is to ensure that all clubs are held to the same standards. The inclusion of sustainability principles demonstrates responsiveness to evolving political and societal expectations (75, 76). This process is further reinforced by the recognition that regulation must be systematically reviewed, as various stakeholders continue to exert pressure on the governance system to accommodate diverse interests, reflecting the growing importance of network-based governance approaches (21, 34).

Organizational governance is reflected in the third layer, particularly in the structures and processes at all levels of an organization, not just within boards (36). Governance structures determine who serves on governing bodies and how roles are organized, while governance processes guide member behavior and ensure key outcomes like accountability, stakeholder participation, and transparency (65).

Notable structural elements at the league level include DFL's financial control bodies, such as licensing management and the licensing committee. These bodies assess and monitor clubs’ financial stability and decide on eligibility to compete in the Bundesliga. The effectiveness of these bodies is influenced by several governance principles, including their composition, domain responsibilities, caliber, independence, tenure, and size. While the extensive experience of the senior license manager and the agility of decision-making within a small team suggest effectiveness (53, 77), challenges may arise when club appeals involve the licensing committee, exposing issues such as inadequate qualification requirements and lack of independence. Furthermore, the absence of a specialized body overseeing the league's financial control mechanisms makes these structures vulnerable to power concentration and conflicts of interest. Effective governance, therefore, requires robust control mechanisms, transparency, and the involvement of relevant stakeholders, highlighting the strong interconnections between governance structures and processes (65).

These interconnections are also evident at the club level. Management boards, supervisory bodies, and nomination committees are essential for ensuring compliance with DFL's regulatory regime. The structures need to provide the necessary accountability and control mechanisms to ensure that financial practices within clubs are conducted responsibly. By distributing decision-making responsibilities across multiple levels, these governance structures help prevent the concentration of power and reduce the risk of financial mismanagement. For instance, management boards oversee daily operations, while oversight bodies independently check these activities to ensure alignment with broader organizational goals and regulatory requirements. In this process, internal and external reporting mechanisms play a key role in planning, managing, and monitoring financial stability within clubs. External reporting involves sharing financial information with stakeholders, thereby enhancing the clubs’ accountability and transparency (33). Internal reporting includes management reviews, financial planning, and scenario planning, which help management bodies and departments develop and execute strategic options (78). Nomination committees are critical in selecting qualified individuals for these boards, ensuring that those in leadership positions have the expertise and integrity needed to navigate complex financial environments and effectively liaise with relevant stakeholder groups, such as club members and fans (79, 80).

### 5.2 RQ2: how does DFL's financial stability regulation enhance governance principles at club and league levels?

DFL's self-regulated financial stability regime presents a flexible system, allowing the league and the clubs to adopt solutions that meet their specific needs and enabling quicker adjustments and less bureaucratic interference. This contrasts with more rigid regulation in other European professional soccer leagues, such as DNCG's centralized financial oversight in France (3). The involvement of various stakeholders, including clubs and league authorities, has great potential to foster a cooperative environment that enhances compliance and commitment to financial discipline.

DFL's financial stability regulation enhances governance principles of clubs, in particular by stringent accountability requirements. Clubs must submit various financial documents, including audited financial statements, budget forecasts, and interim financial reports, to DFL. These documents are thoroughly reviewed by DFL's financial control bodies to assess the financial health of each club and to ensure compliance with the league's financial rules. The submission of these documents is not only a formality but also a crucial part of the ongoing financial oversight that helps to identify and address potential financial issues before they escalate. Additionally, DFL has the authority to request further documentation if any concerns arise during the review process. This continuous monitoring helps the league to enforce financial discipline and prevent clubs from engaging in risky financial behavior that could lead to insolvency (17).

DFL's protection fund, sustained by club contributions, embodies the solidarity principle by helping clubs manage short-term liquidity needs. Recognized as a robust measure against insolvency (3), it upholds competition integrity while ensuring clubs remain accountable for their financial management. Clubs that access the fund must repay the amounts to retain their Bundesliga license for the following season. However, with a capital base of €10 million, the fund may be insufficient to cover significant deficits for one or more clubs. While increasing the fund's capital base could be beneficial, negotiating collective contributions often involves balancing the redistribution of league revenues with the principles of solidarity vs. individual club success.

Therefore, it can be concluded that DFL's financial stability regulation is integral to Bundesliga's ability to maintain a stable and financially healthy league, with fewer instances of club insolvencies compared to other European leagues. Since 1995, no club has entered insolvency while playing in the top tier, and only two clubs have declared insolvency in the second (13). However, insolvency is not an uncommon phenomenon in the lower leagues of the German soccer pyramid. Szymanski and Weimar (13) identified 119 cases of insolvency declarations between 1995 and 2018, which suggests that German football is not significantly more stable than their counterparts in England and France, particularly below the top two tiers. The authors attributed the causes of insolvencies mainly to adverse shocks related to deviations from expected sporting performance, which can lead to relegation. Relegation often results in lower match attendance and revenues, exacerbating financial distress.

While DFL's financial stability regime has been effective in preventing insolvencies, its primary focus is on immediate outcomes, ensuring clubs’ financial stability and their ability to compete throughout the current and the following season. Essentially, clubs are motivated to adapt their governance structures and processes to meet the league's regulatory requirements. However, effective governance goes beyond mere compliance; it requires a proactive approach. Therefore, this study suggests that governance adjustments and extensions are necessary for both the league and the clubs to ensure long-term financial stability and sustainable growth.

### 5.3 RQ3: what adjustments or extensions to DFL's financial stability regulation is needed to align with good governance principles at club and league levels?

To enhance financial stability and adaptability in German professional soccer, an integrated regulatory framework with a long-term perspective is necessary at the systemic governance level.

Aligning DFL's regulations with broader guidelines, such as UEFA's Club Licensing and Financial Sustainability framework and DFB's 3rd League regulations, would help mitigate the instability caused by the promotion and relegation system. Currently, differing financial regulations destabilize clubs that are promoted or relegated, forcing them to quickly adapt to new financial rules (81). The 3rd League, often called “the death league” due to its high insolvency rates (13), presents significant financial risks because of its transitional nature between German professional and semi-professional soccer. Clubs relegated from the 2nd Bundesliga to the 3rd League frequently face severe economic challenges, leading them to overspend on player wages to secure re-promotion, where they can expect significantly higher media rights income. Moreover, increasing financial demands, such as infrastructure upgrades, challenge smaller clubs. For instance, SV Rödinghausen e.V. declined potential promotion to the 3rd League in 2020, unable to meet the stricter financial requirements (82). These situations compromise the meritocratic nature of the promotion and relegation system. A harmonized regulatory approach would provide consistent standards across regions and leagues, reducing economic pressures and maintaining competitive balance by ensuring that on-field success leads to promotion.

To ensure long-term financial stability in the league and clubs, it is recommended that incentives play a greater role alongside strict regulation. These incentives could include direct economic rewards or other benefits. For instance, DFL could implement a reward system that provides additional funds from the media redistribution mechanism to clubs demonstrating efficient financial practices, such as compliance with planned budgets, robust reporting, and risk diversification strategies (81, 83). Furthermore, DFL could introduce a sustainability quota as part of its recently established sustainability regulations (38) encouraging clubs to allocate revenue shares toward sustainable projects. Clubs meeting these targets could receive increased media income, promoting both financial responsibility and contributions to the league's sustainability goals.

Beyond financial rewards, DFL could advocate for sustainable investments, encouraging clubs to voluntarily adopt best practices in areas such as community engagement and environmental stewardship. Although not mandatory, aligning with these initiatives could enhance a club's recognition and sponsorship opportunities. These incentive mechanisms would support more sustainable business models, helping clubs diversify their investments and reduce reliance on income tied to sporting success. This approach is particularly important, as research by Gallagher and Quinn (15) indicates that clubs dependent on central distribution linked to sporting success are more vulnerable to financial distress, while those with independent commercial strategies are better positioned to avoid financial crises.

At the organizational governance level, a shift towards long-term financial planning within clubs is essential. The study's results highlight the importance of considering multiple time periods to better allocate income and expenses. However, the inherent unpredictability of the sport—such as promotion, relegation, and competition qualifications—complicates traditional planning approaches. Scenario planning (84) is a crucial tool for addressing these challenges, enabling clubs to prepare for various potential outcomes and develop resilient financial strategies. Clubs must account for potential supply-side changes, such as media rights redistribution and sustainable sponsorships, along with demand-side shifts, including evolving fan behavior and consumption patterns influenced by digital technologies.

A regulatory requirement for long-term financial planning could offer significant advantages. It would encourage clubs to explore a wide range of future possibilities, fostering financial stability and strategic agility. This approach would also promote transparency, providing DFL and other stakeholders with a clearer understanding of a club's potential trajectories. However, such a requirement could pose challenges, particularly for smaller clubs that may find the administrative burden of extensive financial planning and regular reporting overwhelming. A one-size-fits-all approach could also stifle innovation, preventing clubs from tailoring financial strategies to their unique circumstances (78).

To improve structural governance, regulations should focus on the composition of governance boards at both league and club levels, addressing factors like board size, tenure, independence, and expertise. These measures are crucial for reducing risks like power concentration and competing interests.

Ferkins et al. (85) highlight that while long-serving members bring valuable experience, they can also concentrate influence, leading to resistance to new ideas and necessary reforms, particularly in the fast-paced environment of professional soccer. The potential for health-related absences among board and committee members further underscores the need for succession planning, as the loss of key personnel can disrupt operations, especially when these individuals hold implicit knowledge that is difficult to replace (56). To mitigate these risks, the DFL should mandate term limits, term rotation, and succession planning within its regulatory framework for league and club governance structures.

Additionally, adopting independent governance models, particularly for DFL's licensing committee, is recommended. This aligns with McLeod et al.'s (86) research, which suggests that European club-run leagues could benefit from independent governance practices similar to those used in Australian football. While independent board members are well-positioned to make decisions that benefit the entire league, there is a risk they may become disconnected from the specific needs of clubs. A balanced approach, such as including independent professionals like chartered accountants on the licensing committee or establishing an independent advisory board, could help maintain impartiality while ensuring decisions remain grounded in league realities.

The role of the licensing committee also warrants reconsideration. Currently, its involvement is primarily limited to handling club complaints against license management decisions or if conditions are imposed on a club. This raises questions about its effectiveness as a decision-making body. Expanding its role to be more proactive in all licensing decisions, either by complementing the current process or through a new oversight function, could lead to structural improvements that enhance governance and decision-making.

To ensure high-caliber individuals are selected for governance boards at both league and club levels, particularly for mandatory oversight boards, it is recommended to implement a regulatory requirement for a nomination committee. This committee would rigorously scrutinize candidates for their professional qualifications and ethical standing, helping to mitigate risks such as conflicts of interest, corruption, undue influence, and political infighting within soccer organizations. Adopting selection criteria similar to the English Premier League's Owners’ and Directors’ Test (87) could further ensure that candidates meet high professional and ethical standards. These candidates should demonstrate sound judgment, leadership experience, and a commitment to the organization's values and long-term success. These recommendations align with current research (80), which emphasizes the critical role of nomination committees as gatekeepers in shaping board composition. By formalizing the candidate selection process, the committee can balance efficiency with democratic representation while reducing risks associated with homosocial reproduction—where selections are based on personal connections rather than merit—thereby strengthening integrity in sport organizations.

### 5.4 Contributions, limitations, and future research

This study makes a significant contribution to the sport economics and management literature by linking financial stability regulation with governance principles in professional sport, a connection that has not been extensively explored. Drawing on the multi-dimensional nature of governance, this paper argues that financial stability regulation is a vital component of effective governance, integrating political, systemic, and organizational dimensions, as outlined by Henry and Lee (30). By demonstrating that financial regulation operates within a broader governance context and facilitates coordination between stakeholders—clubs, league authorities, and external actors—this study highlights how regulation serves as a systemic governance mechanism. This interplay creates a governance environment where financial stability mechanisms operationalize governance principles, making them enforceable and effective at the organizational level. In doing so, the study fills a gap in governance literature by illustrating that financial regulation is not merely reactive but a proactive force that drives responsible leadership, organizational resilience, and long-term sustainability in soccer organizations. This novel framework advances governance theory by integrating financial stability into the core of governance, providing a theoretical foundation for understanding how financial stability mechanisms can enhance governance practices, both in soccer and potentially in other sports.

The study's findings have important practical implications. First, regulation remains a crucial governance mechanism for sport leagues to influence club behavior, ensuring adherence to standards that promote the overall interests of the sport. Additionally, the study offers valuable insights for leagues and clubs to enhance governance practices by emphasizing transparency, accountability, and sustainability, which can lead to better decision-making and financial discipline. It encourages clubs to adopt long-term financial planning to navigate the uncertainties of professional sport more effectively. Furthermore, the study suggests that sport leagues should integrate incentive mechanisms for good financial practices into their regulatory frameworks, offering rewards or recognition to clubs that meet financial and strategic goals.

However, the study has several limitations. It primarily focuses on German professional soccer, which may limit the generalizability of its findings to semi-professional and amateur soccer, as well as to other sports or countries with different governance and regulatory frameworks. The use of focus group discussions, while providing rich qualitative data, also introduces challenges. For instance, group dynamics could influence individual opinions, leading to a consensus that may not fully reflect diverse perspectives. Despite efforts to monitor this risk, it is an inherent limitation of focus groups and could result in underrepresented dissenting views. Additionally, there was a risk of bias stemming from participants’ affiliations or perspectives. For example, a club representative might have skewed the discussions toward favoring less regulation. Nevertheless, these biases were largely mitigated by conducting two focus groups, each representing four distinct perspectives: involvement or non-involvement in developing and implementing regulations, and whether they were affected or unaffected by the regulations. The authors also intervened occasionally to maintain an objective “bird's-eye view” of the discussions. Furthermore, while the focus groups aimed to explore complex issues related to financial stability and governance, in-depth discussions on certain topics sometimes limited the time available to address other important themes. Although the discussion prompts were designed to guide the conversations, they occasionally led to a narrower focus, sacrificing breadth for depth. This trade-off provided detailed insights but meant that not every aspect of governance and financial stability could be explored in full detail. The study was conducted during the COVID-19 pandemic, which may have influenced participants’ perspectives. The unprecedented challenges of the pandemic likely heightened concerns about financial stability and shaped views on governance and regulation. Finally, the absence of direct input from current DFL operational license management meant their perspectives were not considered, and they were unable to respond to certain claims made by participants.

Future research could expand on the study's findings by exploring several key areas to deepen the understanding of financial stability and governance in professional soccer. One promising avenue is the harmonization of financial regulations across various levels of professional soccer, including national leagues and international competitions. This research could examine how aligning regulatory frameworks—such as those of the DFL, DFB, and UEFA—might mitigate financial instability caused by promotion and relegation dynamics while ensuring consistent governance standards across leagues. Additionally, investigating the effectiveness of different incentive mechanisms within financial regulation could yield valuable insights into promoting financial discipline and sustainable practices among soccer clubs. Future studies might focus on how economic rewards and voluntary initiatives influence clubs’ financial management and governance behaviors, potentially offering models that could be adopted by other sports.

Moreover, there is a need for research on the impact of independent governance models on the effectiveness of financial regulation in professional sport. Comparative studies between leagues with independent boards and those with club-run structures could assess how governance independence affects overall governance quality. Finally, more qualitative research, particularly observational studies, is needed to explore the actual behavior of league and club representatives in engaging with governance principles. By observing decision-making processes and day-to-day operations, researchers can gain deeper insights into the real-world application of governance frameworks and identify potential gaps between formal regulations and actual practices.

## Data Availability

The raw data supporting the conclusions of this article will be made available by the authors, without undue reservation.
